# CT Morphometric Analysis of Ossification Centres in the Fetal Th12 Vertebra

**DOI:** 10.3390/brainsci15111138

**Published:** 2025-10-24

**Authors:** Magdalena Grzonkowska, Michał Kułakowski, Zofia Dzięcioł-Anikiej, Agnieszka Rogalska, Beata Zwierko, Sara Kierońska-Siwak, Karol Elster, Stanisław Orkisz, Mariusz Baumgart

**Affiliations:** 1 Department of Normal Anatomy, The Ludwik Rydygier Collegium Medicum in Bydgoszcz, The Nicolaus Copernicus University in Toruń, 87-100 Toruń, Poland; mariusz.baumgart@cm.umk.pl; 2 Clinical Department of Orthopedics and Traumatology, Jan Biziel University Hospital nr 2 in Bydgoszcz, Nicolaus Copernicus University in Toruń, 87-100 Toruń, Poland; mkulakowski@poczta.fm (M.K.); agnieszka.rogalska@biziel.pl (A.R.); karol.elster@gmail.com (K.E.); 3 Department of Rehabilitation, Medical University of Białystok, 15-089 Białystok, Poland; zofia.dzieciol-anikiej@umb.edu.pl; 4 Laboratory of Physical Activity, Clinical Research Center (CBK), Medical University of Białystok, 15-089 Białystok, Poland; 5 Department of Electroradiology, Faculty of Health Sciences, Collegium Medicum in Bydgoszcz, Nicolaus Copernicus University in Toruń, 85-626 Bydgoszcz, Poland; beata.zwierko@cm.umk.pl; 6 Department of Clinical Pathomorphology, Faculty of Medicine, Collegium Medicum in Bydgoszcz, Nicolaus Copernicus University in Toruń, 85-094 Bydgoszcz, Poland; sara.kieronska@cm.umk.pl; 7 Department of Normal and Clinical Anatomy, Faculty of Medicine, University of Social Sciences in Łódź, 90-113 Łódź, Poland; orkisz.stanislaw@gmail.com

**Keywords:** fetus, ossification center, vertebral body, thoracic vertebra

## Abstract

Objectives: The present study aimed to determine the growth dynamics of the ossification centers of the twelfth thoracic vertebra in the human fetus, focusing on detailed linear, surface, and volumetric parameters of both the vertebral body and neural processes. Methods: The investigation was based on 55 human fetuses (27 males, 28 females) aged 17–30 weeks of gestation. High-resolution low-dose computed tomography, three-dimensional reconstruction, digital image analysis and appropriate statistical modeling were used to obtain detailed morphometric measurements. Results: All measured morphometric parameters of the Th12 vertebral body ossification center—transverse and sagittal diameters, cross-sectional area, and volume—increased linearly with gestational age (R^2^ = 0.94–0.97). A similar linear growth pattern was demonstrated for the length, width, cross-sectional area, and volume of the right and left neural process ossification centers (R^2^ = 0.97–0.98). No statistically significant sex-related or side-related differences were found, allowing the establishment of single normative growth curves for each parameter. Conclusions: This study provides the first comprehensive CT-based normative data for the ossification centers of the fetal Th12 vertebra in the second and early third trimesters. The presented linear growth models and reference values may assist anatomists, radiologists, obstetricians, and pediatric spine surgeons in estimating fetal age, and in the prenatal and postnatal assessment of congenital spinal anomalies, especially at the thoracolumbar junction. Further research on larger and broader gestational cohorts is warranted to validate and extend these findings.

## 1. Introduction

Congenital vertebral malformations are among the most frequently diagnosed abnormalities of the skeletal system. Analyses of registry data from 2000–2023 demonstrate an increase in their prevalence from approximately 1.2 to 2.2 per 10,000 pregnancy outcomes, including live births, spontaneous miscarriages, and medically indicated terminations [1,2]. This upward trend is attributed both to environmental factors—such as folic acid deficiency, maternal smoking, and obesity—and to advances in prenatal diagnostics that enable the detection of previously unrecognized subclinical forms [2].

The development of the vertebral column during the embryonic and fetal periods is a multistage process involving mesodermal cell migration, somitic segmentation, and focal ossification. The first ossification nuclei appear around the 9th–10th week of gestation. Their morphology and growth dynamics serve as sensitive indicators of normal skeletal development [3,4]. Evaluation of these structures is of both morphological and clinical importance. Disturbances in the ossification process may indicate the presence of isolated anomalies or generalized skeletal dysplasias, such as hemivertebra, vertebra impar, spina bifida occulta, or transitional vertebrae [5,6,7,8,9].

Early detection of spinal malformations is crucial for prognosis, perinatal management, surgical planning, and genetic counseling. Although 2D and 3D ultrasonography remain the primary screening tools, their sensitivity for detecting skeletal dysplasias is only 40–60%. This sensitivity is further reduced in cases with complex anatomical defects, oligohydramnios, unfavorable fetal position, or high maternal body mass index [10]. Magnetic resonance imaging is valuable for evaluating the spinal cord; however, it provides limited resolution for small ossification centers, particularly in fetuses younger than 20 weeks of gestation [3,4,11].

Low-dose fetal computed tomography has introduced a significant breakthrough in prenatal diagnostics. Iterative reconstruction algorithms enable the generation of isotropic voxels smaller than 0.5 mm at a dose of 1–3 mSv, which remains within the recommended safety limits for prenatal imaging [10]. Studies have demonstrated that CT increases the sensitivity for detecting subtle defects of vertebral bodies and arches by 30–40% compared with ultrasonography alone. Despite the minor risk associated with ionizing radiation, this technique is endorsed by the International Society of Ultrasound in Obstetrics and Gynecology (ISUOG) as a second-line investigation when results may influence clinical management [10,12,13,14]. In postmortem studies, micro-CT has proved particularly valuable, as it enables the acquisition of ultra–high-resolution images of small specimens [15,16,17].

Previous studies have focused primarily on the qualitative assessment of vertebral ossification centers [18,19,20]. Quantitative analyses, however, have been limited to selected vertebrae, such as C7 [21] and S1 [22]. To date, no quantitative data have been published for the twelfth thoracic vertebra that would provide normative reference values for its ossification centers across successive gestational weeks.

The present study investigates the ossification process of the body and neural processes of the human fetal twelfth thoracic vertebra using computed tomography. The specific objectives were to:

- examine potential sex-related and side-related differences in all analyzed parameters;
- conduct a quantitative analysis of Th12 ossification centers including linear, surface, and volumetric parameters in order to establish normative reference values for successive gestational weeks;
- determine the growth dynamics of the examined parameters and develop mathematical models that best describe the observed relationships.

## 2. Materials and Methods

### 2.1. Examined Sample

The study material comprised 55 human fetuses (27 males and 28 females) aged between 17 and 30 weeks of gestation. The specimens originated from cases of spontaneous miscarriage and premature delivery and were collected before the year 2000. All preparations are preserved in the collections of the Department of Normal Anatomy, Collegium Medicum in Bydgoszcz, Nicolaus Copernicus University in Toruń, Poland. Ethical approval for the study was granted by the Bioethics Committee of the Ludwik Rydygier Collegium Medicum in Bydgoszcz (decision no. KB 275/2011). All procedures were conducted in full accordance with applicable legal regulations and within the framework of the institutional Body Donation Program for adults and fetuses. The research was carried out in strict adherence to the principles of the Declaration of Helsinki.

Morphometric investigations were conducted between April and June 2025 at the Department of Anatomy, Ludwik Rydygier Collegium Medicum, Nicolaus Copernicus University in Toruń, Poland. Only fetuses with normal anatomical structures, intact tissues, and complete clinical documentation were included in the study. The exclusion criteria comprised congenital malformations, evidence of intrauterine growth restriction, and any other significant musculoskeletal pathology.

Gestational age was determined based on crown–rump length (CRL) and the date of the mother’s last menstrual period. Only cases in which both methods demonstrated high concordance (correlation coefficient R = 0.98, *p* < 0.001) were included in the analysis. This approach ensured precise gestational dating and maintained the homogeneity of the study group [21]. A detailed characterization of the study material, including the number of fetuses and their distribution by gestational age and sex, is presented in Table 1. 

### 2.2. Morphometric Measurements and Assessment of Ossification Centers

**Figure 1:**
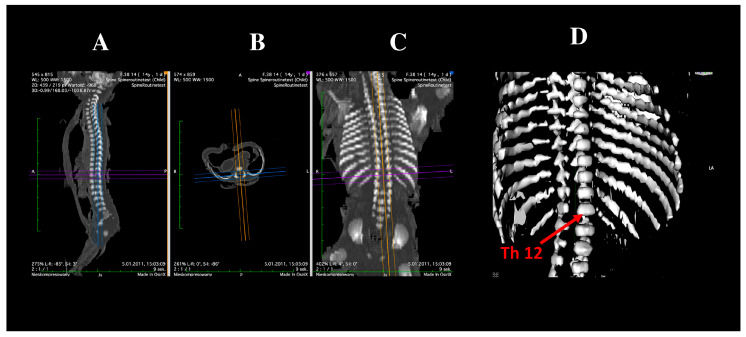
Multiplanar reconstructions (MPRs) of the Th12 vertebra in sagittal (**A**), transverse (**B**), and frontal (**C**) projections, and 3D reconstruction (**D**).

Imaging was performed using a Siemens Biograph mCT 128 computed tomography scanner (Siemens Healthcare GmbH, Erlangen, Germany) at the Department of Positron Emission Tomography and Molecular Imaging, Collegium Medicum, Nicolaus Copernicus University in Bydgoszcz, Poland. Image reconstruction was carried out in DICOM format, generating transverse slices at 0.4 mm intervals (Figure 1).

The range of gray-scale values in Hounsfield units (HUs) extended from −275 to −134 HU for the minimum values and from +1165 to +1558 HU for maximum values. These corresponded to a window width (WW) of 1404–1692 HU and a window level (WL) of +463 to +712 HU. The detailed imaging parameters were as follows: tube current, 60 mAs; tube voltage, 80 kV; pitch, 0.35; field of view (FoV), 180 mm; and gantry rotation time, 0.5 s. The CT data parameters included a slice thickness of 0.4 mm, an image increment of 0.6 mm, and a reconstruction kernel of B45f (medium).

Morphometric measurements of the ossification centers of the Th12 vertebral body and neural processes were performed according to a predefined measurement protocol. For each fetus, detailed analyses were carried out to determine the linear diameters, cross-sectional areas, and volumes of the ossification centers within both the vertebral body and neural processes. Despite the presence of cartilaginous tissue, precise morphometric assessment was possible because the boundaries of the ossifying structures were clearly delineated and could be reliably identified on CT images [21,23,24].

**Figure 2:**
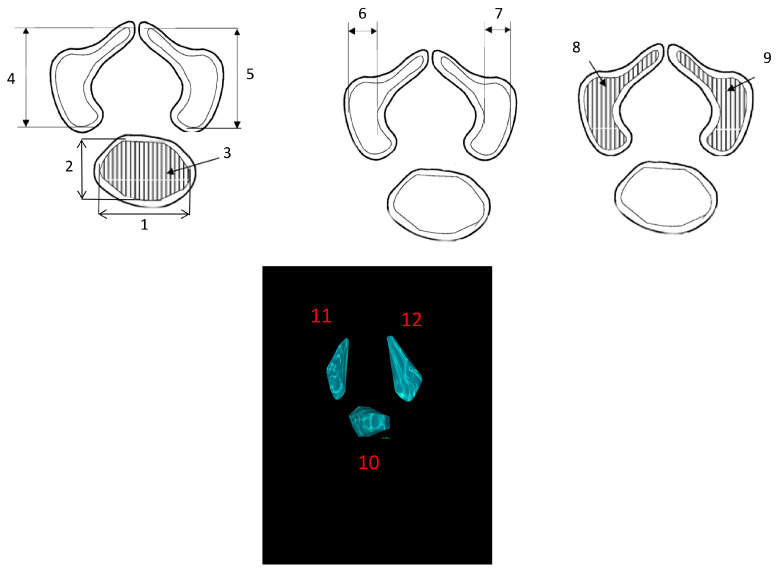
Diagram illustrating the measurement protocol for the ossification centers of the Th12 vertebra.

Measured variables:

1. Transverse diameter of the vertebral body ossification center—defined as distance between the left and right borders of the center in the transverse plane (Figure 2);
2. Sagittal diameter of the vertebral body ossification center—measured as the distance between the anterior and posterior borders of the center in the transverse plane (Figure 2);
3. Cross-sectional area of the vertebral body ossification center—calculated by tracing its outline in the transverse plane (Figure 2);
4. Length of the right and left neural process ossification centers—defined as distance between the proximal and distal borders of each center in the transverse plan (Figure 2);
5. Width of the right and left neural process ossification centers—measured in the transverse plane (Figure 2);
6. Cross-sectional area of the right and left neural process ossification centers—determined by tracing their outlines in the transverse plane (Figure 2);
7. Volume of each ossification center—computed using advanced diagnostic imaging software that enables three-dimensional reconstruction based on spatial position and tissue X-ray attenuation (Figure 2).

### 2.3. Statistical Analysis

Statistical analyses were performed using Statistica version 12.5 (StatSoft Inc., Tulsa, OK, USA) and PQStat version 1.6.2 (PQStat Software, Poznań, Poland). Prior to the analysis of numerical data, the distribution of variables was evaluated using the Shapiro–Wilk test (W), and the homogeneity of variances within comparison groups was assessed using Fisher’s F-test.

Comparisons of morphometric parameters between sides (left vs. right) were performed using the paired Student’s *t*-test, whereas sex-related differences were analyzed with the independent Student’s *t*-test. For variables meeting the assumptions of normality and homogeneity of variances, a one-way analysis of variance (ANOVA) was applied, and significant intergroup differences were further examined using Tukey’s post hoc test. When the assumptions for parametric testing were not met, the nonparametric Kruskal–Wallis test was used as an alternative.

Growth dynamics of the analyzed structures were characterized using regression analysis, which included both linear and nonlinear models. The goodness of fit of each regression model to the empirical data was evaluated using the coefficient of determination (R^2^). Statistical significance was set at *p* < 0.05. Relationships between quantitative variables were assessed using Pearson’s linear correlation coefficient (r).

Each measurement was performed three times under identical technical conditions, and the mean of these three values was used for statistical analysis. As presented in Table 2, the intraclass correlation coefficients (ICCs) estimated from repeated measurements performed by a single investigator (M.G.) indicated excellent reliability (*p* < 0.001).

## 3. Results

Mean values and standard deviations of all morphometric parameters of the ossification centers of the Th12 vertebral body and neural processes across the analyzed gestational period are summarized in Table 3, Table 4 and Table 5. Statistical analysis showed no significant sex-related or side-related differences. Therefore, a single growth curve was constructed for each parameter.

### 3.1. Morphometric Parameters of the Ossification Center of the Th12 Vertebral Body

The developmental dynamics of all examined parameters of the Th12 vertebral body ossification center followed a linear pattern, indicating a direct proportional increase with advancing fetal age.

**Figure 3:**
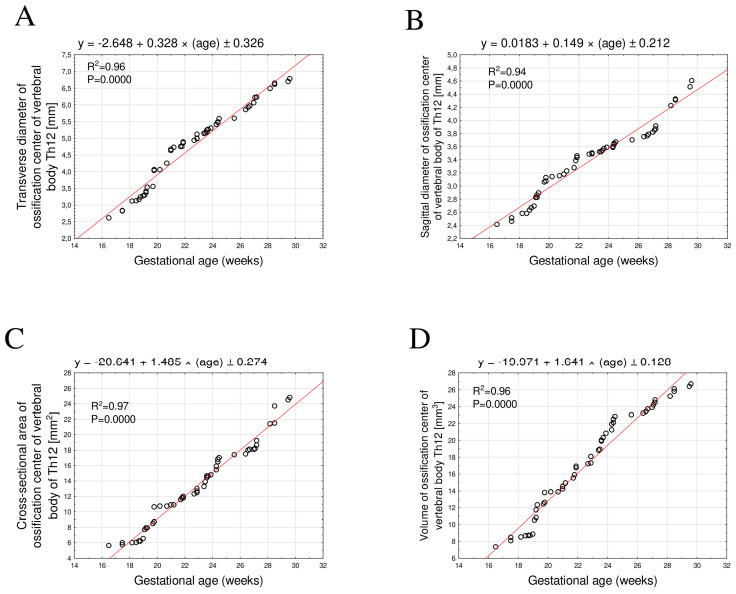
Regression lines for transverse diameter (**A**), sagittal diameter (**B**), cross-sectional area (**C**), and volume (**D**) of the ossification center of the Th12 vertebral body.

The mean transverse diameter of the Th12 vertebral body ossification center in the age between 17 and 30 weeks ranged from 2.61 mm to 6.82 ± 0.11 mm. This relationship was best described by the linear equation y = −2.648 + 0.328 × age ± 0.326; R^2^ = 0.96 (Figure 3A).

The mean sagittal diameter ranged from 2.41 mm to 4.67 ± 0.14 mm and followed a linear growth pattern described by the equation y = 0.0183 + 0.149 × age ± 0.212; R^2^ = 0.94 (Figure 3B).

The mean cross-sectional area increased from 5.60 mm^2^ to 25.08 ± 0.53 mm^2^, showing a linear relationship with fetal age expressed by the equation y = −10.641 + 1.485 × age ± 0.274; R^2^ = 0.97 (Figure 3C).

The mean volume increased from 7.32 mm^3^ to 26.78 ± 0.30 mm^3^ and exhibited a directly proportional growth described by the equation y = −19.971 + 1.641 × age ± 0.128; R^2^ = 0.96 (Figure 3D).

### 3.2. Morphometric Parameters of the Right and Left Th12 Neural Process Ossification Centers

The developmental dynamics of the length, width, cross-sectional area, and volume of the right and left neural process ossification centers of the Th12 vertebra also followed a linear pattern, indicating a direct proportional relationship with fetal age.

**Figure 4:**
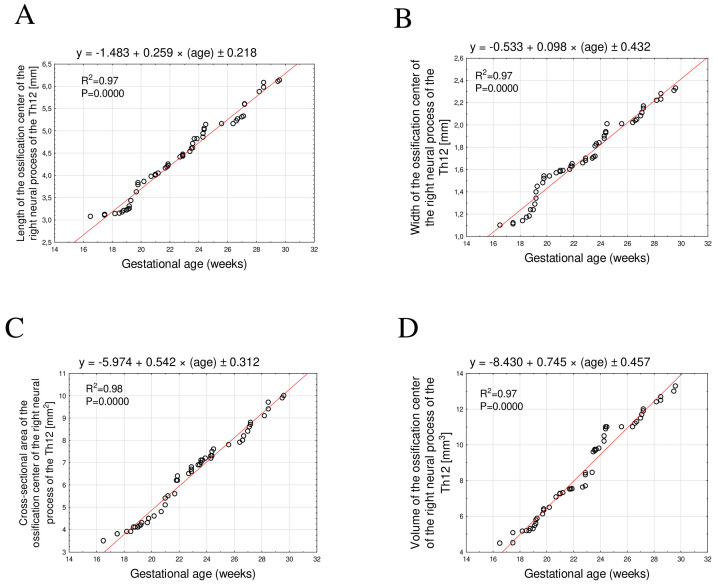
Regression lines for length (**A**), width (**B**), cross-sectional area (**C**), and volume (**D**) of the ossification center of the right Th12 neural process.

**Figure 5:**
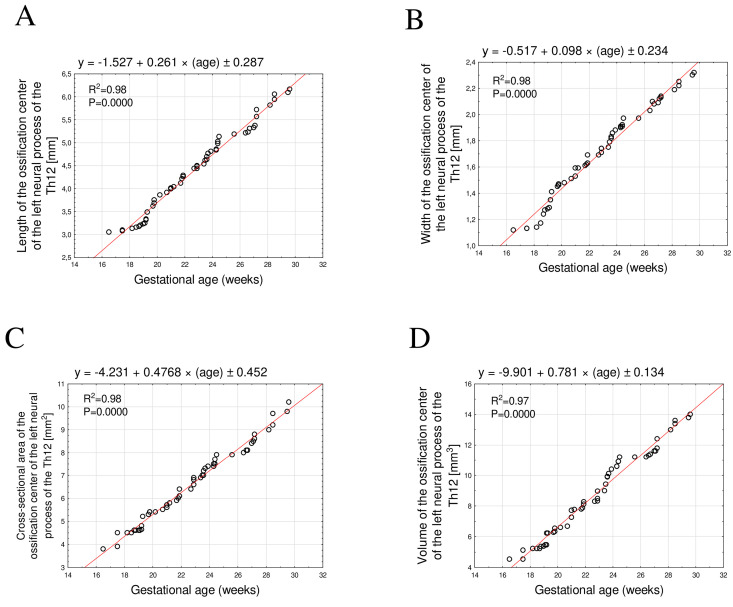
Regression lines for length (**A**), width (**B**), cross-sectional area (**C**), and volume (**D**) of the ossification center of the left Th12 neural process.

The mean length of the Th12 neural process ossification center between 17 and 30 weeks of gestation increased from 3.08 mm to 6.16 ± 0.04 mm on the right and from 3.05 mm to 6.17 ± 0.06 mm on the left. These relationships were best described by the following linear equations y = −1.483 + 0.259 × age ± 0.218; R^2^ = 0.97 for the right side (Figure 4A) and y = −1.527 + 0.261 × age ± 0.287; R^2^ = 0.98 for the left side (Figure 5A).

The mean width of the Th12 neural process ossification center ranged from 1.10 mm to 2.34 ± 0.03 mm on the right and from 1.12 mm to 2.32 ± 0.02 mm on the left. These relationships followed a linear growth pattern described by the equations y = −0.533 + 0.098 × age ± 0.432; R^2^ = 0.97 for the right side (Figure 4B) and y = −0.517 + 0.098 × age ± 0.234; R^2^ = 0.98 for the left side (Figure 5B).

The mean cross-sectional area increased from 3.50 mm^2^ to 10.30 ± 0.41 mm^2^ on the right and from 3.80 mm^2^ to 10.18 ± 0.29 mm^2^ on the left. The corresponding linear functions were y = −5.974 + 0.542 × age ± 0.312; R^2^ = 0.98 for the right side (Figure 4C) and left: y = −4.231 + 0.4768 × age ± 0.452; R^2^ = 0.98 for the left side (Figure 5C).

Finally, the mean volume increased from 4.48 mm^3^ to 13.40 ± 0.34 mm^3^ on the right and from 4.53 mm^3^ to 14.10 ± 0.26 mm^3^ on the left. These relationships followed linear functions described by the equations y = −8.430 + 0.745 × age ± 0.457; R^2^ = 0.97 for the right side (Figure 4D) and y = −9.901 + 0.781 × age ± 0.134; R^2^ = 0.97 for the left side (Figure 5D).

## 4. Discussion

In the present study, we demonstrated that the developmental dynamics of all examined parameters of the ossification centers of both the Th12 vertebral body and neural processes follow a linear model, indicating a directly proportional increase with advancing gestational age. The mean transverse and sagittal diameters, cross-sectional area, and volume of the Th12 vertebral body ossification center, as well as the length, width, cross-sectional area, and volume of the neural process ossification centers, showed a strong correlation with fetal age (R^2^ = 0.94–0.98). Importantly, no significant sex-related or lateral differences were observed, which allowed the construction of uniform growth curves for each parameter. These findings are consistent with the observations of Szpinda et al. [25,26], who likewise reported neither asymmetry nor sexual dimorphism in the process of axial ossification in normal fetuses. At present, there is no robust evidence that fetuses with congenital spinal anomalies exhibit consistent, reproducible sex- or side-dependent morphometric differences in vertebral ossification centers. Most available reports instead address sex epidemiology and the anatomic level or type of defect rather than lateralized morphometrics, underscoring the need for targeted comparative studies against normative datasets [27].

The development of vertebral ossification centers is a complex process that includes the phases of somitogenesis (4–6 weeks of gestation), chondrification (8–9 weeks), and subsequent ossification, which progresses along the cranio-caudal axis [28]. Primary ossification centers first appear in the vertebral bodies and later in the neural processes, with fusion of these structures occurring only after birth [21,29]. Numerous studies have reported variability in the timing and dynamics of ossification [18,26,30]. Nevertheless, the development of ossification centers generally proceeds in a symmetrical and predictable manner [29]. The cross-sectional analysis conducted by Szpinda et al. [26,27], encompassing vertebral bodies and neural arches from C1 to S5, confirms an overall linear increase in areas and volumes, while also demonstrating a gradual decline in absolute values toward the lumbar region, which reflects the functional differentiation of individual spinal segments.

Szpinda et al. [29], using computed tomography to study the Th6 vertebra, measured the parameters of the ossification centers of its body and neural processes. The ossification center of the Th6 vertebral body showed logarithmic growth in transverse and sagittal diameters, described by the equations y = −14.784 + 6.115 × ln(age) ± 0.458 (R^2^ = 0.81) and y = −12.065 + 5.019 × ln(age) ± 0.315 (R^2^ = 0.87), respectively. Its cross-sectional area and volume increased proportionally with age according to the functions y = −15.591 + 1.200 × age ± 1.470 (R^2^ = 0.90) and y = −22.120 + 1.663 × age ± 1.869 (R^2^ = 0.91). The neural process ossification centers on the right and left sides showed the following growth patterns: y = −15.188 + 6.332 × ln(age) ± 0.629 (R^2^ = 0.72) and y = −15.991 + 6.600 × ln(age) ± 0.629 (R^2^ = 0.74) for length, y = −6.716 + 2.814 × ln(age) ± 0.362 (R^2^ = 0.61) and y = −7.058 + 2.976 × ln(age) ± 0.323 (R^2^ = 0.67) for width, y = −5.665 + 0.591 × age ± 1.251 (R^2^ = 0.86) and y = −11.281 + 0.853 × age ± 1.653 (R^2^ = 0.78) for cross-sectional area and y = −9.279 + 0.849 × age ± 2.302 (R^2^ = 0.65) and y = −16.117 + 1.155 × age ± 1.832 (R^2^ = 0.84) for volume.

In our study, ossification of the Th12 vertebra followed a linear pattern, whereas in Th6 a more complex, multimodal growth model was observed. These differences may reflect varying maturation rates between the mid-thoracic and lower thoracic spinal segments, which should be considered when developing growth standards and interpreting early deviations in prenatal diagnostics.

A novel aspect of the present study is the detailed morphometric analysis of the Th12 vertebra as a transitional element, encompassing linear, surface, and volumetric parameters derived from computed tomography. Until now, normative quantitative data for this vertebral level have been lacking, hindering clear differentiation between normal and pathological development. These preliminary data fill an existing gap by offering initial reference values relevant to prenatal diagnostics, especially in borderline cases where ultrasonographic findings remain inconclusive.

The use of computed tomography proved essential for achieving high measurement precision. Owing to its superior spatial resolution and capability for three-dimensional reconstruction, CT enables accurate evaluation of both linear and volumetric parameters [10,18], resulting in highly reliable and reproducible reference standards. With the advancement of imaging technology, including the introduction of micro-CT and the growing availability of scanners, radiologists have shown increasing interest in these techniques—particularly in postmortem fetal examinations within forensic radiology and pathology. In these fields, detailed fetal scanning can support assessment of fetal condition and may, in the future, serve as an alternative to traditional autopsy in determining the cause of death or detecting congenital malformations [11,16,17].

A comparison of ossification center growth rates between normal and anomalous vertebral development would represent an interesting direction for future research. Such an analysis, however, was not feasible in the present study, as the material included only fetuses with normal anatomy. Nevertheless, the reference values established here provide a valuable benchmark for future studies involving pathological cases, enabling objective evaluation of deviations in morphometry and ossification dynamics. The literature emphasizes that quantitative growth models can substantially aid in distinguishing physiological ossification delay from true developmental abnormalities [18,30,31,32,33]. Consequently, the normative data presented here may serve as a foundation for further comparative research and for improving prenatal diagnostic methods that employ advanced three-dimensional imaging and automated image analysis.

The developed growth models also have potential predictive value in the early detection of developmental anomalies. Incorporating high-precision morphometric data into prenatal imaging analysis may enable earlier recognition of abnormalities within the thoracolumbar junction, before they become morphologically apparent. In the future, these models could contribute to artificial intelligence-based diagnostic support systems that automatically identify cases requiring further evaluation based on deviations from reference standards. Although our data should be regarded as preliminary, the obtained nomograms may serve as a starting point for future research on early diagnosis and patient qualification for appropriate therapeutic management.

### Limitations of the Study

A key limitation of this study is the relatively narrow gestational age range of the examined cases (17–30 weeks). In particular, the number of cases from the third trimester was too limited to permit a meaningful comparison of growth dynamics between the second and third trimesters. Specifically, the third-trimester data encompassed only a three-week gestational interval within our study group, which was insufficient to establish reliable separate growth models. Furthermore, all fetal specimens originated from collections obtained before the year 2000, which may limit the representativeness and clinical applicability of the findings. Consequently, the presented nomograms should be regarded as preliminary. A multicentre study covering earlier embryonic stages, the late third trimester, and a broader ethnic spectrum would help strengthen the external validity of the results. Longitudinal correlations with neonatal and infant imaging are likewise warranted to determine how early deviations from the proposed growth curves translate into postnatal pathology [21].

## 5. Conclusions

1. In the studied population of human fetuses aged 17–30 weeks, no significant sex-related or lateral differences were found in the morphometric parameters of the ossification centers of the Th12 vertebral body or neural processes.
2. All analyzed linear, surface, and volumetric dimensions of the Th12 ossification centers exhibited directly proportional growth with fetal age. The growth dynamics followed a linear pattern with a very high degree of regression fit (R^2^ ≥ 0.94).
3. The preliminary normative values obtained for the body and neural processes of the Th12 vertebra represent the first detailed reference data for this anatomical level within the analyzed gestational age range. These data may aid in fetal age estimation and serve as valuable support for ultrasonographic and tomographic evaluation of congenital spinal anomalies, particularly within the thoracolumbar junction. Further studies involving larger and more diverse populations, including both earlier and later prenatal stages, are required to fully validate these observations and to determine their clinical applicability.

## Figures and Tables

**Table 1:** Age, number and sex of the fetuses studied.

Month	GA (Weeks)	Crown-Rump Length (mm)				N	Sex	
		Mean	SD	Min.	Max.		♂	♀
V	17	115	–	115	115	1	0	1
	18	133.3	5.77	130	140	3	1	2
	19	149.5	3.82	143	154	8	3	5
	20	161	2.71	159	165	4	2	2
VI	21	174.8	2.87	171	178	4	3	1
	22	185	1.41	183	186	4	1	3
	23	197.6	2.61	195	202	5	2	3
	24	208.7	3.81	204	213	9	5	4
VII	25	214	–	214	214	1	0	1
	26	229	5.66	225	233	2	1	1
	27	237.5	3.33	233	241	6	6	0
	28	249.5	0.71	249	250	2	0	2
VIII	29	253	0	253	253	2	0	2
	30	263.3	1.26	262	265	4	3	1
Total						55	27	28

**Table 2:** Intra-class correlation coefficient (ICC) values for inter-observer recurrence.

Parameter of the Body Ossification Center of Vertebra Th12	ICC
Transverse diameter	0.995 *
Sagittal diameter	0.996 *
Cross-sectional area	0.998 *
Volume	0.996 *
**Parameter of the Right Ossification Center of the Neural Process of Th12**	
Length	0.996 *
Width	0.996 *
Cross-sectional area	0.997 *
Volume	0.996 *
**Parameter of the Left Ossification Center of the Neural Process of Th12**	
Length	0.997 *
Width	0.997 *
Cross-sectional area	0.997 *
Volume	0.996 *

Inter-class correlation coefficients marked with * are statistically significant at *p*  <  0.0001.

**Table 3:** Morphometric parameters of the ossification center of the Th12 vertebral body.

Month	GA (Weeks)	N	Ossification Center of the Vertebral Body Th12							
			Transverse Diameter (mm)		Sagittal Diameter (mm)		Cross-Sectional Area (mm^2^)		Volume (mm^3^)	
			Mean	SD	Mean	SD	Mean	SD	Mean	SD
V	17	1	2.61	–	2.41	–	5.6	–	7.32	–
	18	3	2.92	0.16	2.52	0.06	5.9	0.17	8.33	0.25
	19	8	3.3	0.13	2.75	0.12	7.04	0.87	10.02	1.5
	20	4	3.92	0.25	3.1	0.04	9.63	1.19	13.18	0.78
VI	21	4	4.57	0.22	3.19	0.03	10.85	0.1	14.38	0.43
	22	5	4.81	0.07	3.39	0.08	11.8	0.16	16.25	0.66
	23	5	5.03	0.09	3.5	0.02	12.76	0.4	17.9	0.66
	24	9	5.32	0.13	3.58	0.04	15.21	1	20.84	1.19
VII	25	1	5.58	–	3.67	–	17	–	22.8	–
	26	2	5.72	0.18	3.73	0.04	17.45	0.07	23.1	0.14
	27	5	6.11	0.14	3.83	0.05	18.37	0.46	24.08	0.52
	28	2	6.46	0.05	4.07	0.22	20.4	1.41	25.1	0.14
VIII	29	2	6.63	0.03	4.32	0.01	22.6	1.56	25.95	0.21
	30	4	6.82	0.11	4.67	0.14	25.08	0.53	26.78	0.3

**Table 4:** Morphometric parameters of the right ossification center of the Th12 neural process.

Month	GA (Weeks)	N	Right Ossification Center of the Neural Process of Th12							
			Length (mm)		Width (mm)		Cross-Sectional Area (mm^2^)		Volume (mm^3^)	
			Mean	SD	Mean	SD	Mean	SD	Mean	SD
V	17	1	3.08	–	1.1	–	3.5	–	4.48	–
	18	3	3.12	0.02	1.12	0.02	3.83	0.06	4.91	0.35
	19	8	3.25	0.09	1.29	0.1	4.13	0.12	5.45	0.26
	20	4	3.78	0.1	1.52	0.03	4.48	0.13	6.34	0.17
VI	21	4	4.01	0.03	1.58	0.01	5.2	0.32	7.23	0.11
	22	5	4.21	0.04	1.63	0.02	6.1	0.35	7.53	0.01
	23	5	4.46	0.05	1.68	0.02	6.7	0.16	8.1	0.41
	24	9	4.83	0.16	1.84	0.08	7.18	0.18	10.13	0.55
VII	25	1	5.14	–	2.01	–	7.6	–	11	–
	26	2	5.16	0	2.02	0.01	7.85	0.07	11	0
	27	5	5.39	0.17	2.1	0.05	8.45	0.31	11.6	0.32
	28	2	5.77	0.16	2.22	0.01	9.05	0.07	12.3	0.14
VIII	29	2	6.03	0.07	2.26	0.04	9.55	0.21	12.6	0.14
	30	4	6.16	0.04	2.34	0.03	10.3	0.41	13.4	0.34

**Table 5:** Morphometric parameters of the left ossification center of the Th12 neural process.

Month	GA (Weeks)	N	Left Ossification Center of the Neural Arches of Th12							
			Length (mm)		Width (mm)		Cross-Sectional Area (mm^2^)		Volume (mm^3^)	
			Mean	SD	Mean	SD	Mean	SD	Mean	SD
V	17	1	3.05	–	1.12	–	3.8	–	4.53	–
	18	3	3.1	0.03	1.13	0.01	4.3	0.35	4.96	0.37
	19	8	3.27	0.11	1.3	0.07	4.69	0.22	5.57	0.41
	20	4	3.73	0.1	1.47	0.01	5.38	0.05	6.42	0.16
VI	21	4	3.99	0.06	1.56	0.04	5.65	0.13	7.35	0.51
	22	5	4.22	0.07	1.64	0.04	6.1	0.22	8.01	0.22
	23	5	4.48	0.04	1.73	0.03	6.72	0.22	8.61	0.34
	24	9	4.8	0.14	1.87	0.05	7.33	0.23	10.3	0.5
VII	25	1	5.13	–	1.97	–	7.9	–	11.2	–
	26	2	5.2	0.01	2	0.04	7.95	0.07	11.2	0
	27	5	5.42	0.19	2.11	0.02	8.42	0.28	11.68	0.39
	28	2	5.77	0.08	2.18	0.02	8.95	0.07	12.8	0.28
VIII	29	2	6	0.08	2.24	0.02	9.45	0.35	13.5	0.14
	30	4	6.17	0.06	2.32	0.02	10.18	0.29	14.1	0.26

## Data Availability

The original contributions presented in this study are included in the article. Further inquiries can be directed at the corresponding author.
